# Toxicity of Infiltrative Lidocaine in Dermatologic Surgery: Are Current Limits Valid?

**DOI:** 10.5826/dpc.1104a120

**Published:** 2021-10-01

**Authors:** Allison Wang, Solomiya Grushchak, Subuhi Kaul, Patrick K Lee, Jerry Feldman

**Affiliations:** 1 Division of Dermatology, Cook County Health, Chicago, IL, USA; 2 Department of Dermatology, University of California, Irvine, California, USA

**Keywords:** lidocaine, anesthesia, tumescent, drug reaction, lidocaine-associated systemic toxicity, Mohs surgery

Local infiltrative lidocaine is the most common anesthetic method used in dermatologic surgery. According to the Food and Drug Administration, the maximum dose of infiltrative lidocaine with and without epinephrine is 7mg/kg and 4.5mg/kg in adults, respectively [1]. Data analyzing the validity of the proposed dosages is lacking, yet these recommendations are widely accepted. We aim to review the origin and stead-fastness of the maximum doses of lidocaine for infiltrative anesthesia in cutaneous surgery.

The maximum lidocaine doses are manufacturer recommendations based on early studies and there are no randomized trials available to validate this regulation [1]. Moreover, these recommendations do not consider the route of administration. Lidocaine was synthesized in 1943, it demonstrated a lethal dose of 1g/kg with subcutaneous infiltration of 0.5% in rodents [2]. In 1949, a human trial performed on over 800 patients showed 500 mg and 1000 mg of lidocaine (with and without epinephrine, respectively) to be safe. 2 cases of systemic toxicity occurred after deliberate overdose with 1.35 g and 3 g of lidocaine in thoracic surgeries. Both patients experienced stupor and convulsions, which improved with intravenous barbiturate [2]. Comparable serum lidocaine levels were confirmed via different routes and doses: 500 mg by epidural administration, 600 mg in brachial plexus blocks, and 1,000 mg in subcutaneous infiltration in legs [1]. The maximum dose limits tend to err on the side of caution and are often inadequate for brachial plexus or epidural blocks and are discussed in anesthesia literature [1,3].

Lidocaine is eliminated via the hepatic cytochrome P450 3A4 pathway [1]. Serum lidocaine concentration depends on several factors including patient and injection method. Severe liver dysfunction or medications that inhibit cytochrome P450 3A4, such as, itraconazole, erythromycin, amiodarone, cimetidine, valproate, may theoretically result in toxicity at lower doses [1, 4]. A study on intravenous administration of lidocaine showed a lower clearance in patients with Child C cirrhosis and concluded that a 50% reduction in dosage was necessary in these patients [5]. Further studies showed that dose reduction is only indicated with repeated blocks administered less than 5 half-lives apart or with continuous infusion, as lidocaine accumulates in cirrhotic patients [1]. Notably, plasma levels attained after subcutaneous injection are lower than that by intravenous injection, thus adjustment of dose may not be necessary after subcutaneous injection, especially if only a single dose is required [1].

Infiltration with epinephrine (potent vasoconstrictor) or as a diluted solution, as in tumescent anesthesia (increased local hydrostatic pressure), slows lidocaine uptake with resulting increased half-life and lower peak plasma levels, that permit a higher maximum dose to be administered [1,4,6]. Lidocaine is lipophilic and slower absorption allows a depot-like effect [4]. Ramon et al studied the combination of epinephrine and dilute lidocaine (0.33%) in patients undergoing facelift surgery. The half-life of lidocaine was found to be increased to 6 hours (compared with 2 hours for intravenous lidocaine) when used together with epinephrine [6]. In addition, researchers were able to safely use 3 times the threshold limit of maximum recommended lidocaine doses (17 to 26 mg/kg of lidocaine). Despite the much higher doses used, the maximum lidocaine plasma concentration remained well below the toxic range (1.14 to 2.2 μg/mL) and no adverse effects were noted [6]. Similarly, doses up to 55mg/kg have been administered safely with tumescent anesthesia [7].

The clinical features of lidocaine overdose depend on serum concentration and different sources cite varying serum concentrations at which toxicity is first observed [3,4,6,8]. Mild symptoms are reported to occur above 5μg/mL. These include dizziness, lightheadedness, and circumoral paresthesias for which observation is recommended. Neurologic and cardiopulmonary complications such as seizures, cardiac and respiratory arrest may occur between doses of 9 to 20μg/mL – in these cases, there is the need for an emergency management with oxygen supplementation and administration of benzodiazepines (with or without intravenous lipid emulsion) [3,4,6,8,9].

In dermatologic surgery, lidocaine associated systemic toxicity (LAST) events are infrequent. In a review of LAST data from 2010 to 2014, only 2 of 67 cases were dermatologist performed procedures, both after topical lidocaine/prilocaine application [10]. Based on a mandatory adverse-event data of office-based surgery in Florida and Alabama, only 4 dermatologist associated complications were reported over 10 years and 1 complication over 6 years, respectively [11]. 3 of these 5 were associated with lidocaine infiltration: the first was transient atrial fibrillation 2 hours after an excision performed with minimal local lidocaine, another was a wrong surgical site during a Mohs procedure, and lastly, a post-excision infection with *Staphylococcus* [10]. Additionally, Alam et al sought to determine if lidocaine injection in the head and neck led to higher absorption. Despite using 48 mLs of 1% lidocaine with 1:100,000 epinephrine, the highest recorded serum level in this study was 0.3 μg/mL [3].

A blanket cap on maximum lidocaine dose in dermatologic surgery is not scientifically valid. Although simple procedures rarely require these high doses, procedures such as Mohs surgery often require stacking of local anesthesia given the multiple layers and the larger areas requiring anesthesia for closure. In addition, various patient factors such as height, weight, race, medical problems should be considered in future studies. When preparing for dermatologic surgery, it would be beneficial to emphasize appropriate anesthetic technique to ensure prevention of intra-arterial injection and confirm the absence of liver disease and/or drugs that may complicate lidocaine elimination when screening patients for procedures.
